# The Perceptions of Potential Prerequisites for Artificial Intelligence in Danish General Practice: Vignette-Based Interview Study Among General Practitioners

**DOI:** 10.2196/63895

**Published:** 2025-03-12

**Authors:** Natasha Lee Jørgensen, Camilla Hoffmann Merrild, Martin Bach Jensen, Thomas B Moeslund, Kristian Kidholm, Janus Laust Thomsen

**Affiliations:** 1 Center for General Practice at Aalborg University, Department of Clinical Medicine, Aalborg University, Selma Lagerløfs vej 249, Aalborg, 9260 Gistrup, Denmark, 45 29807944; 2 Visual Analysis and Perception Lab, Department of Architecture, Design, and Media Technology, Aalborg University, Aalborg, Denmark; 3 Centre for Innovative Medical Technology, Odense University Hospital, Odense, Denmark

**Keywords:** general practice, general practitioners, GPs, artificial intelligence, AI, prerequisites, interviews, vignettes, qualitative study, thematic analysis

## Abstract

**Background:**

Artificial intelligence (AI) has been deemed revolutionary in medicine; however, no AI tools have been implemented or validated in Danish general practice. General practice in Denmark has an excellent digitization system for developing and using AI. Nevertheless, there is a lack of involvement of general practitioners (GPs) in developing AI. The perspectives of GPs as end users are essential for facilitating the next stage of AI development in general practice.

**Objective:**

This study aimed to identify the essential prerequisites that GPs perceive as necessary to realize the potential of AI in Danish general practice.

**Methods:**

This study used semistructured interviews and vignettes among GPs to gain perspectives on the potential of AI in general practice. A total of 12 GPs interested in the potential of AI in general practice were interviewed in 2019 and 2021. The interviews were transcribed verbatim and thematic analysis was conducted to identify the dominant themes throughout the data.

**Results:**

In the data analysis, four main themes were identified as essential prerequisites for GPs when considering the potential of AI in general practice: (1) AI must begin with the low-hanging fruit, (2) AI must be meaningful in the GP’s work, (3) the GP-patient relationship must be maintained despite AI, and (4) AI must be a free, active, and integrated option in the electronic health record (EHR). These 4 themes suggest that the development of AI should initially focus on low-complexity tasks that do not influence patient interactions but facilitate GPs’ work in a meaningful manner as an integrated part of the EHR. Examples of this include routine and administrative tasks.

**Conclusions:**

The research findings outline the participating GPs’ perceptions of the essential prerequisites to consider when exploring the potential applications of AI in primary care settings. We believe that these perceptions of potential prerequisites can support the initial stages of future development and assess the suitability of existing AI tools for general practice.

## Introduction

The research and development of artificial intelligence (AI) has progressed rapidly in recent years and has been deemed revolutionary in medicine. However, AI is still in its early phases of validation and implementation in all health care fields [1,2]. AI tools have predominantly been developed for hospital settings but are also believed to hold transformative potential in general practice, despite fewer AI tools being developed in this setting [3,4].

Denmark is a leading country in the digitization of health care systems [5]. Today, all Danish general practices use digital technology to handle electronic health records (EHRs), send prescriptions to pharmacies and referrals to hospitals, and receive laboratory analysis results and hospital discharge letters. Furthermore, emails and video consultations have become available [6,7]. Owing to this advanced digitization and the collection and management of data in Danish general practice, it is reasonable to believe that AI can be used at some level. In a review of 2023, identifying digital solutions for decision support in Danish general practice, only 1 study dealt with AI [8]. This study described an AI tool for the identification of liver disease [9]. Since the publication of the abovementioned review, an additional study has reported an AI model that predicts cancer risk in patients referred from general practice based on routine blood tests [10]. None of the 2 AI tools reported in the identified studies are currently implemented in general practice. Thus, based on the identified evidence, research in this area is limited, with no validation, implementation, or application of AI tools currently available in Danish general practice.

When developing new technology, including potential end users and other stakeholders is essential [11]. This is defined as the exploration phase, an important step in the implementation process, creating awareness of the existing issues that need attention and potential improvement [12]. Contradictorily, the involvement of general practitioners (GPs) in developing concrete AI tools for general practice is limited [13,14]. In addition, limited research has been conducted to clarify the qualitative perspectives of GPs and other stakeholders regarding AI in general practice [15-20]. A closer examination of the same research reveals that only 3 studies have exclusively addressed the perspectives of GPs [18-20]. The research shows that GPs and other stakeholders generally have a positive attitude toward AI [15-18,20], but the practical and ethical implications of applying AI in general practice need clarification. This includes privacy, liability, and financial considerations [15]. Multiple uses of AI are envisioned in general practice, particularly within tasks of an administrative character [16,17]. This is due to concerns about replacing human competencies with AI [15,18,19]. Finally, it is essential to consider co-creation and collaboration with end users to ensure optimal adoption of AI in general practice [16,17,20].

Thus, when considering the development of AI in Danish general practice, it is necessary to understand the contexts and perspectives of the end users. Therefore, this study aimed to explore and define the prerequisites for applying AI in general practice from the GPs' perspective. GPs can provide insights into the essential conditions that must be met to realize the potential of AI in this setting. Establishing these foundational requirements will support the next steps toward advancing AI development in Danish general practice.

## Methods

### Qualitative Approach

This study applied an inductive approach using semistructured interviews and vignettes to iteratively explore and provide detailed insights into the GPs’ perspectives on the prerequisites for AI in general practice. The vignette method was chosen to exemplify hypothetical scenarios of AI in general practice and create meaningful engagement with the participating GPs, as no AI tool has been implemented and validated in Danish general practice. Vignettes are typically used in qualitative research as sociological tools to study beliefs, attitudes, values, and perceptions [21-23]. The authors followed the Standards for Reporting Qualitative Research (SRQR) [24].

### Researcher Characteristics and Reflexivity

The first author is a PhD student studying user perspectives on AI in general practice. The second author, an associate professor of anthropology, specializes in qualitative methods and perspectives of patients and GPs. The third author is a professor and a GP focused on integrating new technology as a support tool in general practice. The fourth author is a professor who specializes in responsible and value-driven AI. The fifth author, a professor of health economics, concentrates on health care innovations in medical technology. The final author is a professor and a GP studying the potential of new technology in general practice. All the authors believe that ethically applied and legally compliant AI can benefit general practice.

### Context

Denmark is a welfare state with universal health care that ensures free and direct access to primary health care for all residents. The health care system is organized into 5 administrative regions, with general practice serving as a gatekeeper and the first point of contact [6]. The study was conducted in the North Denmark Region.

### Sampling Strategy

Purposive sampling [25] was applied to recruit participants who were characterized as “first adopters” [26]. Therefore, the GP network was used to recruit GPs associated with the Center for General Practice at Aalborg University (CAM AAU) and consultants affiliated with the Quality Unit for General Practice in the North Denmark Region (Nord-KAP). We categorized GPs associated with these organizations as “first adopters” within their profession, as they typically demonstrate a distinctive interest in research in new medicine and technology. Thus, by sampling these specific GPs, we ensured satisfactory AI literacy for the interviews when discussing viewpoints on AI in general practice.

The study’s first author, who also served as the only interviewer, had no personal or professional relationship with the participants, which might have influenced the results. Although the third and last authors were acquainted with several respondents, they did not know who provided specific statements. This ensured that any possible casual acquaintances had minimal influence on the analysis or conclusions of the study.

### Ethical Considerations

Owing to the nature of the study, ethical approval was deemed unnecessary under Danish law (Section 14, Subsection 2 of of the Danish Act on Committees) [27]. This decision was based on legal provisions and confirmed through consultation with the institutional guidelines. According to Danish law, ethical approval is not required for qualitative interview studies that do not involve human biological materials or sensitive personal health data. However, written informed consent was obtained from all participants. To ensure privacy and confidentiality, all data were pseudonymized. Participants received no compensation for their participation in the interviews.

### Data Collection Methods and Instruments

Interviews were conducted in Danish and face-to-face at the general practice clinics. The interviews lasted 35 to 95 minutes and were recorded using a dictaphone. Each interview began with introducing the respondents to the study and its purpose. The respondents were then presented with 5 individual vignettes, followed by questions from a semistructured interview guide. All vignettes depicted scenarios concerning a female patient presenting in general practice where different forms of AI are applied. The first vignette aimed to initiate a discussion on the understanding of data and AI to create a common frame of reference between the interviewer and the respondent. The following vignettes (numbers 2‐5) aimed to clarify the GPs’ point of view on AI in general practice, first on AI used in tasks concerning automation and then on AI applied to tasks related to decision support. The GPs were presented with a generic case and a more specific example for each type of AI task. The interview guide consisted of open-ended questions and prompted the exploration of GPs’ perspectives on their experiences with new technology and AI in general practice, challenges and benefits, and their overall attitudes toward technology and AI in health care. The vignettes applied in this study are found in (Multimedia Appendix 1).

### Units of Study

A total of 12 GPs participated in this study. In total, 9 semistructured interviews were conducted in 2019, and the remaining 3 were conducted in 2021. In addition, 6 men and 6 women participated in the study. At the time of the interviews, participants’ ages ranged from 36 to 66 years, with a mean age of 47.7 years. Their professional experience as GPs varied from 2 to 30 years, with an average of 11 years. This diversity in age and experience closely reflects the general Danish GP population [28].

### Data Processing and Analysis

For data processing, all collected interview data were stored safely on a file share server administered by Aalborg University in line with the obligations of the European Union’s General Data Protection Regulation (GDPR). All the data were pseudonymized when uploaded to the secure file share with a key for safe reference storage.

In addition, 2 student assistants were assigned to transcribe all interview data verbatim for subsequent analysis. The student assistants were verbally instructed and provided written guidelines for the assigned tasks.

NVivo 14 (QSR International) coding software was used for all data analysis. Thematic analysis guided by this study’s research question was conducted on the collected interview data [29]. The analysis commenced with familiarization with the data by reading and rereading the transcripts and listening to the interview audio recordings. Initial insights and analytical ideas were noted while reading and listening to the data. Data coding was then initiated, which involved systematically coding the interview transcripts and identifying themes through an iterative process that prioritized transparency and rigor. All coded data were then re-read to collate the data relevant to each code. The initial themes were generated based on the developed codes. Each theme was then reviewed and refined to align with the study objective, resulting in a final set of themes. The authors have translated the quotes stated in this paper from Danish to English.

### Techniques to Enhance Trustworthiness

A pilot test was conducted with 2 GPs before the primary data collection to enhance the validity and reliability of the vignettes and the interview guide. The pilot test evaluated the clarity, relevance, and comprehensibility of the vignettes and the appropriateness of the interview questions. Feedback provided by the GPs during this process was systematically incorporated to refine and improve the vignettes and interview guide, ensuring that they effectively addressed the study objectives and were suitable for target participants.

Although purposive sampling was used to ensure that participants were selected based on their relevance to the research objectives, efforts were made to achieve diversity among participants. This included considering variations in age, gender, and years of professional experience. Striving for diversity enhances the robustness of the findings by ensuring a broader range of perspectives.

The iterative process of dialogue and consensus building among all the authors validated the findings, strengthened the credibility of the results, and ensured that the final manuscript reflected the collective agreement of the authors.

## Results

**Figure 1.**
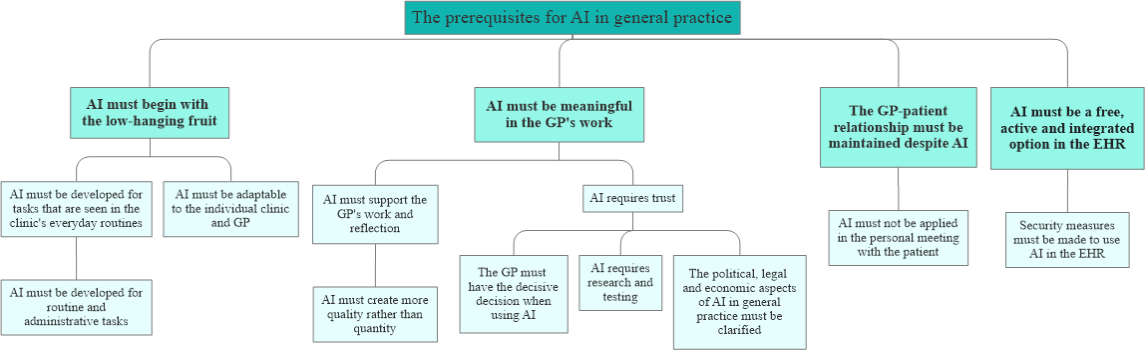
Illustration of the four themes and connected codes defined in the analysis of the interview data. AI: artificial intelligence; GP: general practitioner; EHR: electronic health record.

The analysis of data resulted in four themes: (1) AI must begin with the low-hanging fruit, (2) AI must be meaningful in the GP’s work, (3) the GP-patient relationship must be maintained despite AI, and (4) AI must be a free, active, and integrated option in the EHR. All 4 themes contain codes that support the results of the analysis. Figure 1 shows the hierarchy of themes and codes.

### AI Must Begin With the Low-Hanging Fruit

When GPs were asked to identify the most relevant tasks in general practice with the potential to develop AI, their response was to address “the low-hanging fruit,” which was the first theme of the analysis. GPs defined “the low-hanging fruit” as low-complexity tasks with the necessary data available to develop AI that can facilitate GPs’ work at a practical level, with minimal impact on the GP-patient relationship. The GPs highlighted the importance of developing AI for everyday clinical tasks rather than for rare or highly specialized diagnostic processes that might have limited use. They emphasized that AI should enhance tasks and tools that already perform well in general practice but have the potential for further optimization. A significant opportunity lies in applying AI to routine administrative tasks such as managing medications and processing test results, which are time-consuming but cognitively straightforward. In addition, the GPs suggested exploring the possibility of developing AI for tasks typically handled by practice staff, such as organizing test responses and scheduling annual check-ups, where systematic and repetitive processes dominate. Furthermore, the GPs emphasized that AI would be received constructively if initially introduced for tasks considered “low-hanging fruit,” as this approach would allow them to build confidence in the technology through early practical successes.

The GPs also emphasized that AI must be grounded in tasks embodied in the practices and workflows of individual GPs and clinics. Owing to the individual general practice clinics varying in organization with different workflows and ways of approaching and handling tasks, it is necessary that AI can be adapted to a specific setting.

*... but then again, I just think it is important that you get the opportunity to tailor it to the individual practice, that it does not just become a system that has to fit all clinics because we do it very differently. Some only do an annual check, some don’t do annual checks at all, and some, as we do here, for example, have split it up and made an individual plan for people. It is not certain that you need to come for an annual check once a year; there may well be a large check every two years, but then you come to a smaller check. It has to be something where you can put your own prerequisites on it (AI), but it could be something like when the patient walks out the door, that you say send a message to the patient that they should contact us for the purpose of blood tests or something in six months.*

[Participant No. 7]

### AI Must Be Meaningful in GP’s Work

The second theme is “AI must be meaningful in the GP’s work.” The GPs state that any AI with the potential for application in general practice must be relevant and sensible. Irrelevant information in the EHR becomes an annoyance rather than an aid in the GP’s information-intensive work life.

*There is a lack of reflection like this to say, well, when is this pop-up relevant, and when do we have to be confronted with it? Well, if you had set aside an hour to do a review of automated responses or what do I know, you could perhaps achieve a lot more health in that hour than on the four patients you see in an hour. So, it is a question of where you put your resources.*

[Participant No. 4]

To be meaningful, the GPs emphasize that AI must function as a supportive tool in their work, leveraging its strengths, such as data analysis and processing, to enhance their practice. The GPs also proposed using AI as a sparring partner to support and challenge their professional judgment and create reflection in their practice. Furthermore, Participant No. 3 accentuated that AI should contribute to more quality than quantity in general practice to achieve “less useless work and more useful work.” The GPs anticipate that introducing AI into general practice will likely lead to a redistribution of tasks. Consequently, more health-promoting activities will be prioritized, but the overall workload will remain high.

*I think it’s an illusion that we’ll get to the point where robots and AI take over our work so that we don’t have to work in the future - because it always brings other things along, but it’s a good idea, as it can give me less useless work and more useful work. That’s fine. But I think that the sum of work, it probably will for my part - and here in the clinic - it will probably be constant regardless of what you do. But it is also because we will probably just admit more patients and more tasks. That is what I imagine.*

[Participant No. 3]

Trust is a critical prerequisite for GPs to find AI meaningful. The GPs emphasized that AI must not function independently but should assist the GP’s work. It is also stated that authority should remain with the GP*,* as fear of AI taking control could lead to mistrust. Consequently, GPs suggest that the data generated by AI should be crosschecked with the GPs’ observations and intuition to ensure that the tasks are performed correctly. In addition, AI must be flawless to benefit GP. The GPs prefer a challenging daily workload, valuing their ability to perform their tasks well rather than on AI for suboptimal results.

*…What you can fear about AI is that it gets its own authority in a way—that the authority goes away—or that the decision goes from being the doctor’s to being the system’s. It is a slippery slope. It’s not either or, it’s on the way to something … Then, it has to be compared with the processes I go through when I sit across from a person who has all kinds of stories and compare them with the data I look at and perhaps all the ones I do not look at, which could be a weakness in my situation, but which can be the strength in the AI’s situation. So this AI will have its strengths, but can also in some ways trump and perhaps blind us to some things we would otherwise have seen if we had sat and talked to the patient instead.*

[Participant No. 4]

Several GPs stated the importance of making conclusive decisions and maintaining awareness of their responsibility rather than unquestioningly trusting AI. Consequently, questions were raised regarding the role of AI if GPs must always oversee and approve the decision-making process. Double-checking multiple outcomes is time-consuming if the GPs have to modify or override the AI-generated results.

Knowledge and familiarity with the AI algorithm were highlighted among the GPs as necessary. Thus, AI requires further research and testing to ensure the trust of GPs. Any AI tool developed for general practice is expected to undergo comprehensive evaluation in research studies to assess both the advantages and disadvantages of the technology. It is also essential to explore the impact of AI on external factors, such as work structures and the organization of general practice, as well as to consider patients’ perspectives on AI tools.

In addition to the research-related and external outcomes of AI in general practice, the GPs emphasize the need to address political, legal, and economic aspects to build trust in the technology. Politically, the GPs stress that the use of AI should be initiated by the GPs themselves, ensuring that AI does not become a tool for political management. AI must be developed and used primarily for the benefit of general practice. and most importantly, for the benefit of patients. The legal implications of AI must be clarified, particularly regarding the distribution of liability in the event of errors. Finally, the GPs expect an investigation into the economic impact of AI in general practice at a societal level.

### The GP-Patient Relationship Must Be Maintained Despite AI

Preserving the GP-patient relationship despite the integration of AI in general practice is the third essential prerequisite. The GPs consider a significant part of their work to occur at the human level, particularly during personal interactions with patients. While GPs see technological tools as beneficial for more standardized procedures such as administrative tasks, they view them as a distancing factor during patient consultations. Patients must not feel that they are interacting with a computer, as personal contact between the GP and the patient remains central to the practice. The GPs clarify that most health-promoting outcomes arise from the time spent with patients, and they would prefer to dedicate time to patient care rather than to computers and other technological aids.

*So, what I am skeptical about is whether the patient feels they have a doctor or whether they feel they have a computer. So, the thing about whether you trust that the doctor knows that something has to be done and all that - I can fear that both I as a doctor will lose that, but also that patients will feel that they are losing it. Thus, the doctor-patient relationship could suffer slightly in such a scenario. However, it could also be that it does not suffer because they’re just smart up there in that hospital, right ... So in that way, I am not so opposed to it, but I have some skepticism about whether you will achieve what you want with it.*

[Participant No. 5]

To preserve the personal relationship between the GP and the patient, the GPs strongly prefer that AI is not present during consultations. Given that consultations typically last only 10 minutes, GPs must carefully manage their time to achieve all objectives, making it challenging to envision how AI can support personal connections with patients. Consequently, they did not foresee the use of AI during consultations. Instead, they suggested using AI to analyze historical data related to the reason for inquiry.

### AI Must Be a Free, Active, and Integrated Option in the EHR

The fourth prerequisite highlighted by the GPs is the seamless integration of AI into existing clinical EHR systems. This integration should allow the AI to function as an optional tool that can be activated and accessed on demand. This ensures that GPs can use AI tools to seek assistance and support for specific tasks. The GPs express strong opposition to any interruptions in the EHR workflow, such as pop-ups, and emphasize the need for complete control over the AI functionality, including the ability to turn it on and off as needed, regardless of the tool’s type or purpose.

A high level of security is another prerequisite for the GPs to adopt AI into EHR systems. The GPs emphasize that information must not be lost as it moves from human oversight to AI processes, nor should it accumulate unnecessarily within the EHR. Robust safety measures are essential to ensure that tasks handled by AI are appropriately followed by GPs or other practice staff, even if the information is not immediately necessary. In addition, a “backstop” mechanism should be integrated into the EHR to collect and safeguard any AI-handled information that has not been acknowledged by a GP or other practice staff, ensuring no data is overlooked or mishandled.

*No, then I wouldn’t like it, because there are many of the test answers, we get here in the house, that are normal, but normal answers can also give rise to some form of action. So, if you imagine that you had a machine that sent normal responses to the patient, something would go wrong. Then we must have a stumbling block in relation to follow-up.*

[Participant No. 6]

## Discussion

### Statement of Principal Findings

This study identified 4 main themes as prerequisites that GPs consider essential for realizing the perceived potential of AI in general practice: (1) AI must begin with the low-hanging fruit, (2) AI must be meaningful in the GP’s work, (3) the GP-patient relationship must be maintained despite AI, and (4) AI must be a free, active, and integrated option in the EHR.

### Strengths and Weaknesses of the Study

In this study, we applied vignettes as a stimulus for reflecting on hypothetical scenarios, given that AI has not yet been implemented or validated in Danish general practice. The vignettes served as a tool to facilitate discussions on topics in which the included GPs generally had limited experience. However, because vignettes are traditionally designed to simulate real-world scenarios, the absence of AI in current practice may reduce their perceived credibility [30].

A key reason for including first adopters associated with CAM AAU and Nord-KAP was to ensure satisfactory AI literacy among participants, addressing the low AI literacy observed in similar studies [15,20,31]. At the outset of the interviews, the participants were asked about their knowledge of AI, and we determined that their AI literacy was sufficient for the study. However, this study has limitations due to purposive sampling and recruitment from a limited geographical area (the North Denmark Region). This may have affected the transferability of the study to other contexts. Despite these limitations, the age and professional experience of the participating GPs provided a reasonable range of diversity, closely resembling that of the general Danish GP population [28]. Therefore, the results of this study should be interpreted in the specific context of data collection and as reflective of the participants’ perceptions [29].

The interview data were collected in 2019 and 2021, and with the rapid technological advancements in AI in recent years, the evidence presented in this study may be outdated. However, because AI has not yet been widely implemented in general practice, the overall development of AI remains less directly relevant to the daily work of GPs. In addition, the core values of general practice have remained unchanged since the time of the interviews [32]. This argument is underlined in the paper by Nassehi and Ramvi [33], which states the continuous importance of care in general practice despite the prevalence of digital technologies. Thus, the insights from this study remain relevant in the context of the ongoing development of AI in general practice.

### Comparison With Previous Work

Several views and attitudes similar to those identified in this study on the prerequisites for AI in general practice can, upon closer examination, be found in previous research [15-20]. Regarding what this study defined as “low-hanging fruit,” referring to starting AI development for low-complexity tasks, other studies have shown comparable preferences, for example, introducing AI to reduce administrative burdens [17,19,20]. For instance, it has been suggested that the daily workload of GPs can be alleviated through preselection and patient prioritization [20].

In addition, the potential of AI to support administrative tasks such as clinical documentation, practice operations, and triage is seen to enhance efficiency. This allows GPs more time and cognitive freedom to focus on the medical and social complexities of patient care [17]. These findings highlight a discrepancy between the needs of GPs and the current dominant research trends in AI, such as diagnostic decision support tools [13]. This further emphasizes the importance of defining the prerequisites for AI, as outlined in this study, to ensure that the core values of general practice are preserved.

This study found that the included GPs are open to the potential of AI to alleviate specific routine tasks without diminishing their primary responsibilities. Other studies also support this view, emphasizing that AI will never fully replace GPs, especially in clinical decision-making [17]. It has been established that AI should act as a support tool, complementing the GP’s clinical judgment while remaining sensitive to the patient’s specific concerns and context [15,20]. Furthermore, 1 study demonstrated that technology could negatively influence GPs’ decision-making, potentially impacting treatment outcomes [20]. Thus, our study findings reinforce that GPs should retain authority to make decisive decisions when working with AI.

Similarly, trust appears to be a crucial factor in AI acceptance of the included GPs. Our findings align with those of other studies that emphasize trust as a key driver of AI adoption and usage by GPs [34]. GPs must be open to AI and confident in the information it provides [15]. However, trust extends beyond GPs; patients’ trust in AI depends on their GP’s trust and acceptance [35]. Therefore, successful integration of AI into general practice depends on the trust of both GPs and patients, ensuring that a human-centric approach is at the core of AI adoption.

Furthermore, the GPs in our study underscored that AI should not detract time from patients. Comparably, previous research indicates that GPs fear that AI may compromise the quality of patient care and undermine the GP-patient relationship [15]. It has also been suggested by previous research that GPs believe that AI lacks the essential human competencies required for patient care [19]. AI is seen as unable to account for the social and economic factors that influence patient care, as the ability to maintain a holistic view of care develops through long-term relationships between individuals and communities [17,19,20]. Therefore, preserving the GP-patient relationship must be a key consideration when developing AI for general practice, underscoring the importance of protecting this relationship in the AI integration process.

Finally, similar to our findings, previous studies have noted that the minimum requirements for AI in EHRs are essential for ensuring optimal use. For example, AI systems should be designed to preserve autonomy, health data privacy, and security within the EHR system [15,20]. GPs must be able to use AI intuitively and issues such as software interoperability, usability, and workflow integration must be addressed to ensure that AI has a meaningful impact on general practice [15,17]. This underscores the importance of minimizing the effort required by GPs to effectively use AI, making it a critical prerequisite for designing AI tools for general practice.

### Possible Mechanisms and Implications of the Study

The GPs included in this study believe that meaningful use of AI in general practice begins with low-complexity tasks, aligning with the earlier definition of “low-hanging fruit.” In this study, we understand “meaningful” as a quality in human-computer interaction [36]. Thus, by realizing AI for administrative and supporting tasks already embedded in everyday clinical routines, AI can support GPs’ actual work and reflections at a practical level.

The results of this study further demonstrate that the GP-patient relationship can be preserved if AI is designed to handle routine and administrative tasks. The included GPs underline their central role in patient interactions, and their suggestion to assign AI to more peripheral tasks further underpins the narrative of GPs as family doctors [37]. However, today’s general practice has evolved significantly from traditional family medicine, particularly regarding patient independence and symptom interpretation [38]. The time and space available to patients are becoming more restrained in modern general practice, yet patient involvement and autonomy are reaching unprecedented significance. Therefore, it is essential to clarify how AI can support the modernization of general practice while preserving the essence of GP-patient relationships.

It has previously been determined that patients trust the use of AI if their GPs trust it [35]. The trust of GPs in this study hinges on understanding the underlying algorithm and making conclusive decisions based on AI outputs. However, this raises the question of whether GPs can trust AI, particularly its current “black box” form. This challenge underscores the importance of exploring the possibility of explainable AI (XAI) [39]. XAI is an emerging research area that should be considered to realize the potential of transparent and trustworthy AI in general practice.

Furthermore, ensuring that AI is an optional and customizable feature within the EHR is critical. Allowing individual clinics to adjust AI tools according to their preferences and routines can improve their adaptability. This will enable GPs to determine the extent to which AI assists in health care work. Thus, modernizing the EHR system is essential for using the AI capacity. This ensures that the GP’s use of AI remains an active and deliberate choice, reinforcing their autonomy and trust in the technology.

To further enhance trust and meaningful AI in general practice, it is essential to establish close collaboration among GPs, EHR suppliers, and other information technology stakeholders when developing and potentially implementing AI. This collaborative synergy would ensure that potential AI tools meet general practice needs while aligning with the upcoming regulatory framework introduced by the European Union, such as the GDPR and AI Act. These frameworks enhance transparency, accountability, and trust in AI systems, thereby supporting their integration into general practice [40,41].

### Unanswered Questions and Future Research

When examining the study’s defined prerequisites for AI in general practice, it becomes evident that the included GPs recognize the transformative potential of AI. Nevertheless, further research is imperative to identify relevant low-complexity tasks of a routine and administrative nature before commencing the development of suitable AI tools. Classifying suitable low-complexity tasks for AI in general practice will require comprehensive effort, but such classification is critical for aligning development with implementation. By identifying tasks suitable for AI in general practice, groundwork can be laid for a seamless transition from design to deployment.

While this study only addressed the exploration phase of implementation [12], it is essential to consider the remaining implementation phases before initiating development [42,43]. Furthermore, it could be favorable to incorporate the study’s prerequisites into early implementation strategies, which could ensure the alignment of new AI tools with GPs’ expectations and perceptions of how AI should function in general practice.

While the study results highlight the importance of addressing political, legal, and economic aspects to build trust in AI, it does not delve into concerns regarding privacy, liability, and financial considerations stated in previous research [15]. Future research exploring these areas within the context of AI in Danish general practice would be highly valuable.

As no AI tool has been implemented or validated in Danish general practice, we believe that the defined prerequisites of the study can guide the initial phases of the future development of AI. It will also be valuable to consider these prerequisites when evaluating new AI tools to determine whether they are suitable for general practice.

## Supplementary material

10.2196/63895 Multimedia Appendix 1 Vignettes applied in the study.

## Abbreviations

AI: artificial intelligence
CAM AAU: Center for General Practice at Aalborg University
EHR: electronic health record
GDPR: General Data Protection Regulation
GP: general practitioner
Nord-KAP: Quality Unit for General Practice in the North Denmark Region
SRQR: Standards for Reporting Qualitative Research
XAI: explainable artificial intelligence
