# Supplementary material for: The Perceptions of Potential Prerequisites for Artificial Intelligence in Danish General Practice: Vignette-Based Interview Study Among General Practitioners
Source: JMIR Med Inform. 2025 Mar 12;13:e63895. doi: 10.2196/63895 (PMC11921986; doi:10.2196/63895)
Supplement: Multimedia Appendix 1 [file medinform-v13-e63895-s001.docx]

# Appendix 1

## Vignette 1

During a consultation with her general practitioner, Annette Jensen is informed about a new project where artificial intelligence is to be developed and tested in the clinic. The IT solution, based on artificial intelligence, will be developed using health data. The purpose of artificial intelligence in the clinic is to serve as a helping hand in the general practitioner's work.

## Vignette 2

A clinic similar to yours is offered an IT solution on a trial basis to help automate part of the general practitioner's daily work, thereby alleviating the workload in the clinic. The IT solution is categorized as artificial intelligence based on algorithms that can, for example, sort and prioritize messages and test results, as well as provide automatic response suggestions.

## Vignette 3

After a long day at the clinic, the general practitioner needs to review test results. As a new feature, the IT system has started generating automatic suggestions for responses and treatments for patients with positive urine cultures. The automatic suggestion is based on algorithms developed using artificial intelligence. If the general practitioner agrees with the response, all that needs to be done is to approve it. The response is then sent to the patient.

## Vignette 4

Later the same clinic gets the opportunity to test an IT solution that can support the diagnosis and treatment of patients. The IT solution is a decision support model created based on algorithms derived from artificial intelligence.

## Vignette 5

Annette Jensen comes to the clinic with pain in the lower abdomen. The general practitioner examines Annette thoroughly but is unsure of the diagnosis. The general practitioner knows that researchers have successfully developed artificial intelligence based on an algorithm that can detect ovarian cancer. The algorithm could support the general practitioner’s diagnosis. The general practitioner can choose to use the algorithm as decision support, potentially advancing Annette's further examination and referral.
